# Chlorine Nuclear Magnetic Resonance as a Sensitive Probe to Study Crystalline to Glasslike Interfaces in Calcium Phosphates

**DOI:** 10.1021/acs.langmuir.6c01029

**Published:** 2026-05-26

**Authors:** Rokas Lemežis, Jonas Stadulis, Audrius Drabavičius, Aleksej Zarkov, Vytautas Klimavicius

**Affiliations:** 1 Institute of Chemical Physics, 54694 Vilnius University, Saulėtekio al. 3, Vilnius LT-10257, Lithuania; 2 Institute of Chemistry, 54694 Vilnius University, Saulėtekio al. 3, Vilnius LT-10257, Lithuania; 3 Center for Physical Sciences and Technology (FTMC), Saulėtekio al. 3, Vilnius LT-10257, Lithuania

## Abstract

The calcium chlorapatite Ca_5_(PO_4_)_3_Cl samples with controlled surface morphology ranging from low to high surface areas were studied using ^35,37^Cl magic-angle spinning and static nuclear magnetic resonance (NMR). Complex ^35,37^Cl NMR lineshapes obtained for all studied Ca_5_(PO_4_)_3_Cl samples, consisting of two components, were analyzed using quadrupolar and Czjzek models that characterize highly crystalline and glasslike moieties, respectively. Transmission electron microscopy micrographs confirmed the formation of the glasslike material as a shell, while crystalline moieties were formed in the core of the particles. The ratio of crystalline to glasslike material was related to the morphology of the samples: smaller particles contained more glasslike species, while larger particles were composed mostly of crystalline material. The results obtained highlighted the importance of quadrupolar NMR as an experimental method for correlating the particle morphology with phase composition at the molecular level. Moreover, quadrupolar NMR demonstrated its robustness as a complementary technique to X-ray diffraction for the characterization of complex materials, especially those consisting of several coexisting phases, some of which lack long-range order, e.g., glasses and glasslike materials.

## Graphical abstract

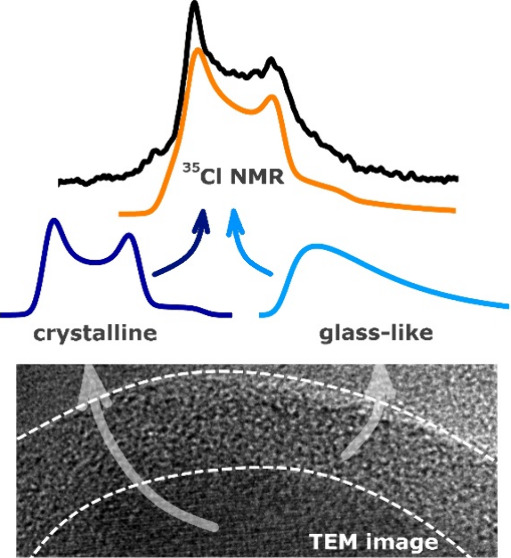

## 1 Introduction

Calcium phosphates (CPs), due to their versatility and resemblance to biological hard tissues, have found numerous applications in medicine, bone regeneration, and drug delivery systems.^1,2^ Moreover, their structural diversity, compositional flexibility, and mechanical and thermal properties stimulate their further application as optical materials, for catalysis and many others.^3,4^ Typically, CPs are classified by their crystal structure, e.g., apatitic, whitlockite-type, pyrophosphates, tricalcium phosphates (TCP), amorphous (ACP), etc., Ca-to-P ratio, or anion (OH^–^, CO_3_^2–^, Cl^–^, and F^–^) present in the crystal structure.^1^ The desired physical and chemical properties are determined by the combination of these structural motifs.

The diversity of synthesis techniques enables the preparation of CPs with varying dimensionality, morphological features, degree of crystallinity, and surface properties.^5,6^ All these parameters are crucial for specific applications. One of the synthetic approaches applicable for the synthesis of phosphate-based materials is the molten salt method.^7,8^ It is a simple and cost-effective technique that enables the preparation of materials spanning from nanodimensional particles to millimeter-scale single crystals.^9–11^ In this approach, the chemical reaction occurs in the melt, which significantly reduces the reaction temperature compared to the conventional solid-state reaction method. Moreover, a similar approach can be used for the preparation of phosphate glasses, which are a wide subfamily of phosphate-based materials.^12^

The composition of CPs is typically well-characterized by Fourier transform infrared spectroscopy (FTIR) and ^31^P nuclear magnetic resonance (NMR), which are sensitive to determining the chemical species present in the CPs.^13^ The gold standard for crystal structure characterization is powder X-ray diffraction (XRD), which is sensitive to slight changes in the crystal structure. Nevertheless, in many cases, more than one phase is present in CPs, which are often amorphous or glasslike, making reliable characterization by XRD a challenging task.^14^ Morphological features are typically characterized using scanning or transmission electron microscopy (SEM and TEM, respectively), which provide insight into the shape and size of the synthesized particles but, unfortunately, lack chemical and structural information at the molecular level. Therefore, an experimental technique or methodology capable of relating the morphology of a sample to its chemical and phase compositions would be highly beneficial.

Solid-state NMR of quadrupolar nuclei (spin quantum number greater than 1/2) enables probing of the electric field gradients and provides valuable information on the local environment around the nucleus, which depends on the structures present in the material. The shape of signals, characterized by the quadrupolar coupling constant *C*_Q_ and the asymmetry parameter η*,* originating from quadrupolar nuclei, is extremely sensitive even to slight structural changes or the degree of crystallinity.^15–18^ On the other hand, noncrystalline materials, such as glasses or glasslike materials, that lack long-range but remain short-range order are well-characterized by the Czjzek model that includes the distribution of the electric field gradient (EFG) tensors.^19^ The Czjzek and similar models were previously used to describe ^35^Cl NMR spectra of the MgCl_2_ catalyst support and F/Cl-apatite solid solutions.^20,21^

In this paper, we address the challenging task of correlating the morphological features of CP powder with their structures at a molecular level by utilizing ^35,37^Cl NMR. The calcium chlorapatite Ca_5_(PO_4_)_3_Cl samples with controlled surface morphology ranging from low to high particle size and thus different surface areas were chosen. By using advanced analysis of ^35,37^Cl NMR spectra, we aim to highlight the boundaries between the highly crystalline and glasslike species.

## 2 Experimental Section

### 2.1 Synthesis and Characterization

The calcium chlorapatite (Ca_5_(PO_4_)_3_Cl) powders were synthesized according to previously reported procedures involving the treatment of amorphous calcium phosphate (ACP) with potassium chloride (KCl, >99%) and calcium chloride (CaCl_2_, ≥94%) salts as a flux.^22^ For the synthesis, ACP powders were mixed with salts, resulting in an ACP:flux mass ratio of 1:2 or 1:10, where KCl and CaCl_2_ salt molar ratios were 9:1, 8:2, 7:3, and 6:4. The obtained mixtures were heat-treated at *T* = 750, 900, 1000, 1100, or 1200 °C for 5 h. After the reaction, the obtained products were washed with hot deionized water to remove the residual salts. The remaining products were dried in an oven at 100 °C.

Powder X-ray diffraction (XRD) analysis of the samples was performed using a Rigaku MiniFlex II diffractometer (Cu Kα, λ = 1.5419 Å) working in the Bragg–Brentano (θ/2θ) geometry. The data were collected within the 10–60° 2θ angle range with a speed of 5° min^–1^.

The morphological features of the synthesized powders were analyzed by scanning electron microscopy (SEM) using a Hitachi SU-70 microscope.

Transmission electron microscopy (TEM) measurements were performed using a Tecnai F20 X-TWIN (FEI company) microscope with an accelerating voltage of 200 kV equipped with a Gatan Orius CCD camera. Measurements were performed in the bright-field regime.

### 2.2 Solid-State NMR

Solid-state ^35^Cl and ^37^Cl MAS NMR spectra were measured at 9.4 T on a Bruker Avance III HD NMR spectrometer operating at 39.2 and 32.6 MHz, respectively, using a 4 mm ^1^H-X CP MAS NMR probe. Temperature was stabilized at 298 K, and 10 kHz MAS was used. For the ^35,37^Cl measurements, the Hahn-echo pulse sequence (π/2-delay-π-delay-acquire) was employed using one rotor period as echo delay, 51,200 scans were accumulated, and the recycle delay was set to 1 s. The π/2 excitation pulse was equal to 5 μs.

Static solid-state ^35^Cl NMR spectra were measured using a 5 mm static NMR probe. The temperature was stabilized at 298 K, the π/2 pulse was 4 μs (62.5 kHz nutation frequency), and recycle delay was set to 1 s. For echo NMR spectra measurements, a solid-echo pulse sequence (π/2-delay-π/2-delay-acquire) was used with the following parameters: 50 μs interpulse delay and 20,480 scans. For ^35^Cl QCPMG measurements (π/2-acquire-(π-acquire)_*n*_), a train consisting of 20 refocusing π pulses was used and 32,000 scans were accumulated.

For selected samples, solid-state ^35^Cl MAS NMR measurements were performed at 14.1 T using a Bruker Avance Neo 600 spectrometer operating at 58.8 MHz, equipped with 2.5 mm Trigamma triple resonance MAS probe. The temperature was stabilized at 298 K, and 20 kHz MAS was used. For the Hahn-echo pulse sequence (π/2-delay-π-delay-acquire), the echo delay was set to 10 rotor periods to avoid background signal, the π/2 excitation pulse was 8 μs, 61,440 scans were accumulated, and 1 s recycle delay was used.

The ^35,37^Cl NMR spectra were referenced using an external standard of sodium chloride (NaCl, *δ*(^35,37^Cl) = 0 ppm).

### 2.3 Spectral Analysis

Spectral analysis of ^35,37^Cl MAS and echo NMR spectra was carried out using the MRSimulator (version 1.0.0) open-source Python package.^23^ Spectral fitting was carried out using the LMfit (version 1.3.1) open-source Python package with the least-squares minimization procedure.^24^ To improve the calculation speed, the probability distribution function of the Czjzek model was calculated before the spectral fitting procedure.

At the beginning, the fitting procedure was performed for a representative sample (Supporting Information, Table S1), which allowed us to obtain the spectral fitting parameters set used for subsequent approximations. The spectra were fitted using two spectral components attributed to the standard quadrupolar model, which is the consequence of second-order quadrupolar broadening, and the Czjzek model, where quadrupolar tensor parameters are not singular-valued but subject to a distribution function.^25,26^ Afterward, the quadrupolar (*δ*_iso_, *C*_Q_, and *η*) and Czjzek model’s (*δ*_iso_ and *σ*) parameters were kept fixed while the fitting procedure was run in parallel for the complete set of spectral data sets obtained for each sample. Such an approach allowed us to vary the intensity of lines attributed to the quadrupolar and Czjzek models. In certain cases, the perturbation parameter of the Czjzek model was allowed to vary to better fit the experimental data set. The obtained results, namely, the *R* ^2^, which were in the range of 0.82–0.99, showed the validity of the procedure. ^37^Cl NMR spectral fitting was performed for all the data sets assuming the same or scaled, e.g., *C*_Q_, parameters (Supporting Information, Table S2).

## 3 Results and Discussion

The synthesis of the samples discussed in this work was previously reported.^22^ To summarize the previous study, the selective conversion of ACP into single-phase Ca_5_(PO_4_)_3_Cl or Ca_2_PO_4_Cl was investigated by varying the KCl/CaCl_2_ ratio in the flux, which also resulted in the controlled formation of particles of different sizes and shapes. The analysis of the obtained powders included XRD, SEM/EDX, FTIR, as well as solid-state NMR. The ^31^P MAS solid-state NMR analysis allowed us to confirm the formation of the Ca_5_(PO_4_)_3_Cl and Ca_2_PO_4_Cl phases. Nevertheless, ^31^P MAS solid-state NMR appeared to be less sensitive to the powder morphology than SEM analysis. The variation in FWHM is 26–83 Hz (Supporting Information, Figure S1). The preliminary ^35^Cl MAS NMR spectra, successfully obtained for several samples, have shown their potential for highlighting fine structural features in the samples.

Depending on the K:Ca ratio used in the synthesis, the end powders consisted of calcium chlorapatite (Ca_5_(PO_4_)_3_Cl), goryainovite (Ca_2_PO_4_Cl), or a mixture of both. Ca_5_(PO_4_)_3_Cl, possessing a monoclinic crystal structure (see XRD data in Figures S2 and S3, Supporting Information), features a more symmetric chlorine environment compared to Ca_2_PO_4_Cl, forming an orthorhombic crystal structure.^27^ Even subtle changes in chlorine environments reflect changes to ^35^Cl solid-state NMR powder patterns.^28,29^ Therefore, the Ca_2_PO_4_Cl should exhibit a broader ^35^Cl NMR spectral line, which, to our knowledge, has not been reported previously. The static NMR measurements and piecewise acquisition allowed us to obtain the ^35^Cl echo NMR spectrum for this crystalline material (Figure S4, Supporting Information) with the following parameters: *δ*_iso_ = 106 ppm, *C*_Q_ = 7.9 MHz, and *η* = 0.2. Due to a wide spectral width stretching around 250 kHz at 9.4 T and complicated time-consuming acquisition, only the samples containing the Ca_5_(PO_4_)_3_Cl phase were investigated and discussed further in this manuscript.

The ^35^Cl MAS NMR spectra obtained at 9.4 and 14.1 T, along with representative SEM micrographs, are listed in Figure 1. Measurements were performed at two magnetic fields to confirm the accuracy of measurements, and the fitting procedure as the second-order quadrupolar interaction is inversely proportional to the external magnetic field strength. These spectra are the characteristic examples of the studied samples; a complete set of all studied samples and echo spectra with all the fitting parameters is shown in the Supporting Information (Tables S2–S5 and Figures S5 and S6).

**Figure 1:**
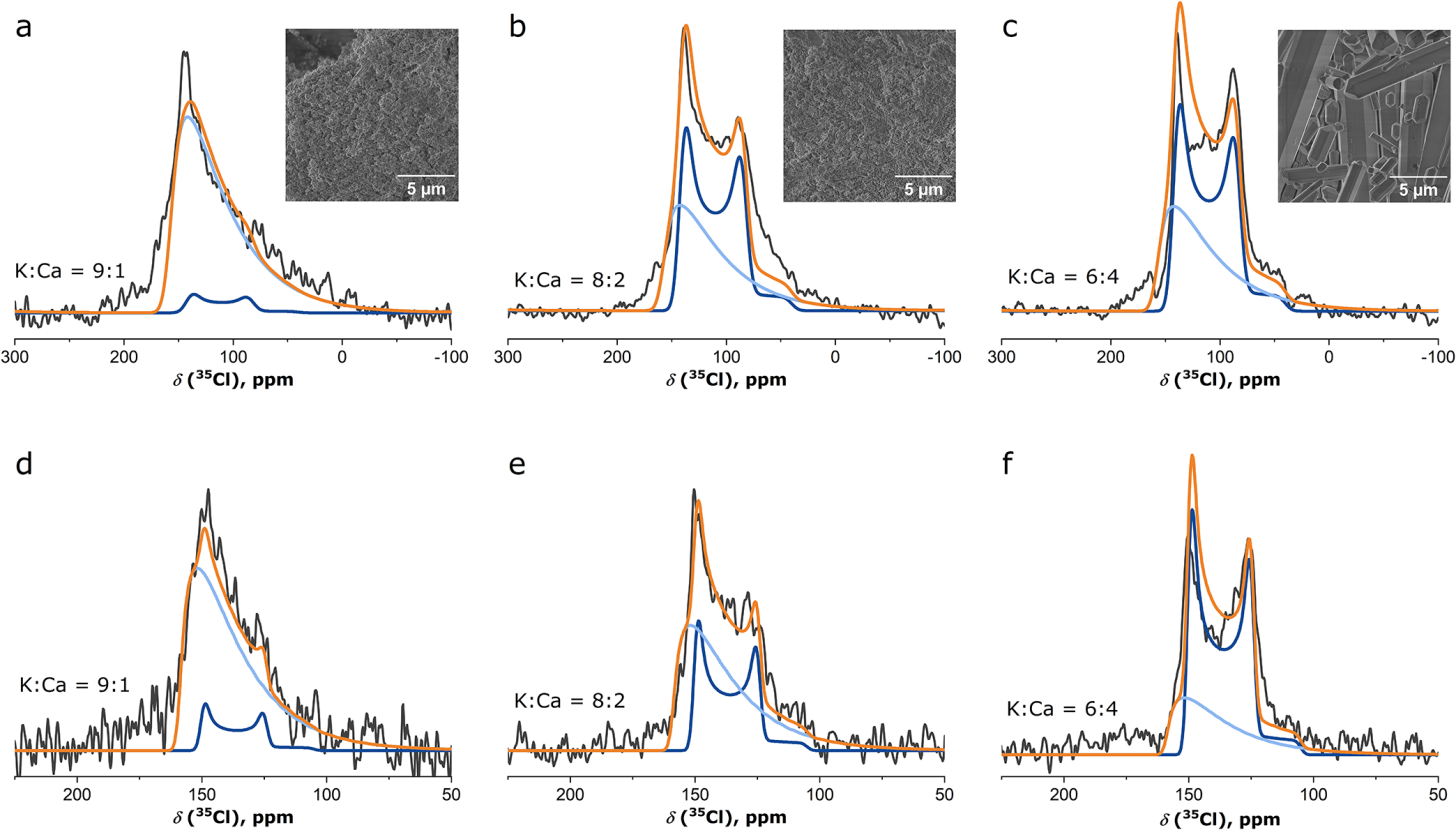
^35^Cl MAS NMR spectra obtained at 9.4 T (a–c) and 14.1 T (d–f) of Ca_5_(PO_4_)_3_Cl synthesized with different molar ratios of KCl and CaCl_2_ (from 9:1 to 6:4, *T* = 750 °C, ACP/flux = 1:2, *t* = 5 h). Experimental data are shown in dark gray, fitted spectral lines: quadrupolar, dark blue; Czjzek model, light blue; cumulative fit, orange. Spectral fitting parameters are given in the Supporting Information. SEM micrographs are shown side by side to the spectra for comparison.

^35^Cl NMR MAS and echo spectra exhibit complex lineshapes. These were fitted using two spectral lines corresponding to two characteristic molecular environments, namely, one featuring the quadrupolar lineshape (*δ*_iso_ = 157 ppm, *C*_Q_ = 1.7 MHz, and *η* = 0) and the second one featuring the lineshape typical to glasslike materials characterized by the Czjzek model that is described by isotropic chemical shift *δ*_iso_ and perturbation parameter *σ* (*δ*_iso_ = 159 ppm,*σ* = 0.4).^26^ Measurements performed at different magnetic fields (9.4 and 14.1 T) provide the same fitting parameters (see the Supporting Information, Tables S3 and S4). Our obtained fitting parameters are in agreement with those previously obtained for solid mixtures of F,Cl apatites.^21^ Quadrupolar lineshapes refer to highly crystalline structural motifs, while phases lacking long-distance order, e.g., glasslike materials, are better described by the Czjzek model, which mimics the disorder present in these materials.^30–37^ Analysis of ^35^Cl data (Figure 1) shows that the change in the K:Ca ratio in the flux from 9:1 to 6:4 results in a larger fraction of the quadrupolar line; in other words, a higher amount of the crystalline phase is formed. Nevertheless, the difference in the K:Ca ratio shows no effect on the fitted lineshape parameters; it only leads to a difference in the amount of the corresponding phases. On the other hand, the K:Ca ratio in the synthesis is strongly related to the morphology of the sample, evident in the SEM micrographs (Figure 1 and Figure S5, Supporting Information). Upon changing the K:Ca ratio from 9:1 to 6:4, the morphology changes drastically from agglomerated submicrometer particles possessing a nearly spherical shape to micrometer-scale uniform rodlike particles. By correlating the changes of sample morphology and tendencies observed in corresponding ^35^Cl MAS and echo spectra, we are able to state that the crystalline phase, resulting in the quadrupolar lineshape in the spectra, is formed as a core, while the glasslike phase is formed as a shell of the particles. Such a hypothesis well-explains the observed ^35^Cl NMR spectral changes (Figure 1); namely, the larger relative surface area of the smaller particles contributes significantly to the chemical species characterized by the Czjzek model, while larger particles having much smaller relative surface area possess ^35^Cl NMR spectra containing lines characterized mostly by the quadrupolar lineshape model. This means that ^35^Cl NMR is sensitive to probe crystalline and glasslike interfaces in such materials.

To gain more insights into the morphology of the Ca_5_(PO_4_)_3_Cl samples, TEM measurements were performed. Obtaining TEM images of apatite samples with glasslike or amorphous structures is a difficult task due to electron-beam-induced crystallization.^38,39^ Although such an effect was also encountered here, it was possible to obtain high-resolution TEM images for the studied systems (Figure 2). It is seen that the core of the particle is composed of the crystalline Ca_5_(PO_4_)_3_Cl phase, while the surface of the particle is formed of a glasslike structure. By comparison of this observation with the SEM data, which indicated that the smallest particles are formed when K:Ca = 9:1 and the largest when K:Ca = 6:4, it is clear that larger particles should have a lower glasslike moiety fraction in the sample. It is important to note that the studied samples range from nearly monodisperse spherical to highly polydisperse rodlike particles, so TEM images should be analyzed qualitatively. These demonstrate the presence of crystalline and glasslike phases within the same particle. Therefore, the tendency shown in Figure 1 seems very logical, that by the increasing size of the Ca_5_(PO_4_)_3_Cl particles, the relative amount of shell glasslike species reduces, resulting in an increase in the ^35^Cl NMR line characterized by the quadrupolar lineshape model.

**Figure 2:**
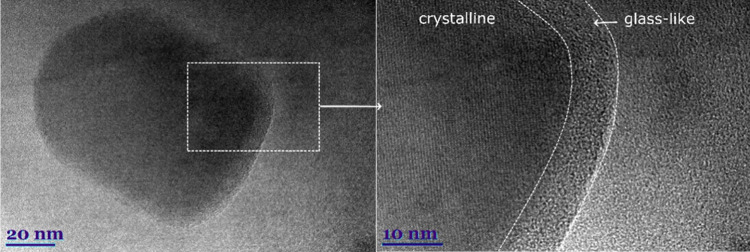
TEM micrographs of the Ca_5_(PO_4_)_3_Cl sample (KCl:CaCl_2_ = 9:1, *T* = 750 °C, *t* = 5 h, ACP:flux = 1:1).

To investigate the robustness of the method related to isotope effects, ^37^Cl MAS NMR measurements were performed for the same samples (K:Ca = 9:1–6:4). Representative spectra are shown in Figure 3, another ^37^Cl MAS NMR spectrum is given in the Supporting Information (Figure S5). Although ^35^Cl is typically an isotope of choice for studying chlorine-containing materials, due to higher natural abundance and higher gyromagnetic ratio; however, ^37^Cl possesses a smaller nuclear quadrupole moment, which results in a reduced quadrupolar coupling constant (*Q*(^35^Cl)/*Q*(^37^Cl) = *C*_Q_(^35^Cl)/*C*_Q_(^37^Cl) = 1.26889).^40^ While ^37^Cl quadrupolar coupling constants are reduced in comparison to ^35^Cl, the asymmetry parameter remains the same, and the spectral peak positions are slightly shifted due to the quadrupole-induced shift.^41^ The detailed fitting data are given in the Supporting Information, Tables S5 and S6. Accounting for the previously mentioned effects, the ^37^Cl data correlate with the ^35^Cl data, namely the fraction of material described by the quadrupolar lineshape increases while that characterized by the Czjzek model decreases depending on the parameter K:Ca (from 9:1 to 6:4) **(** Figure 3). The parameters obtained after the fitting procedure are the following: for the site characterized using the quadrupolar model *δ*_iso_ = 160 ppm, *C*_Q_ = 1.35 MHz, *η* = 0 and for the site characterized by the Czjzek model *δ*_iso_ = 163 ppm, *σ* = 0.33.

**Figure 3:**
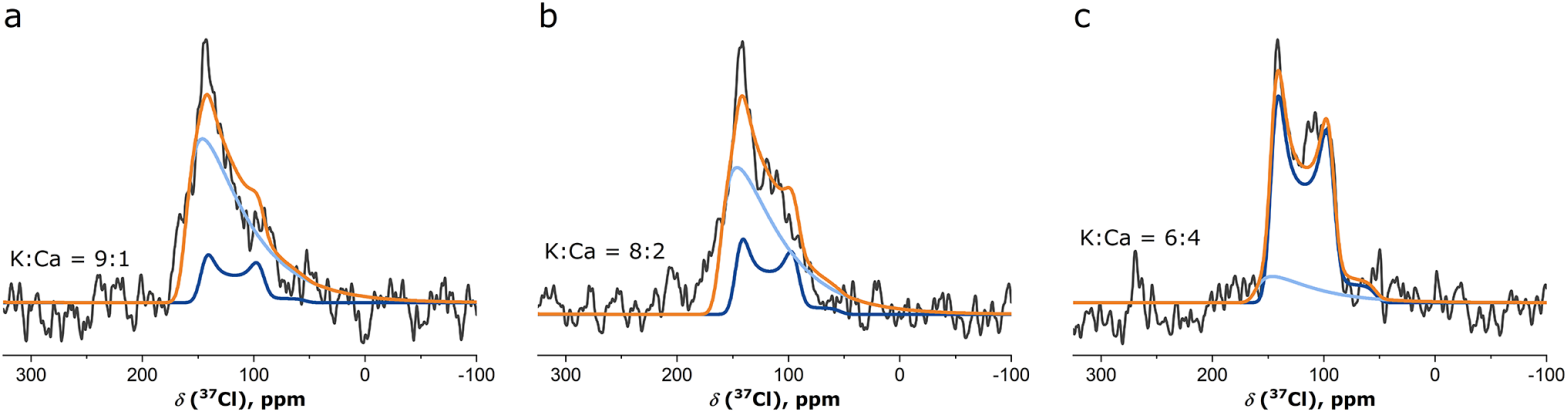
^37^Cl MAS NMR spectra of Ca_5_(PO_4_)_3_Cl synthesized with different molar ratios of KCl and CaCl_2_ (from 9:1 to 6:4, *T* = 750 °C, ACP/flux = 1:2, *t* = 5 h) obtained at 9.4 T. Experimental data are shown in dark gray, fitted spectral lines: quadrupolar, dark blue; Czjzek model, light blue; cumulative fit, orange. Spectral fitting parameters are given in the Supporting Information.

To further investigate the applicability of this model, which allows analyzing the ^35^Cl and ^37^Cl NMR spectral lines depending on the fine structural motifs present in the samples, a set of Ca_5_(PO_4_)_3_Cl samples, prepared with varying ACP:flux ratios (1:1–1:10), were investigated (Supporting Information, Figure S7). For the ^35^Cl MAS NMR data, the fitting procedure described previously was used with the parameters given in the Supporting Information, Tables S3 and S4. All data sets showed the presence of quadrupolar and Czjzek-type lines in ^35^Cl MAS NMR spectra. The sample synthesized with an ACP:flux ratio of 1:10 also showed the presence of a line attributed to KCl salt, whose intensity decreased upon washing with deionized water.^42^ The dependence of the parameter *I*_Quadrupolar_/(*I*_Quadrupolar_ + *I*_Czjzek_), which is the ratio of the integral intensity of the line fitted using the quadrupolar model with the total integral value, on the ACP:flux ratio is shown in Figure 4. It is seen that the parameter *I*_Quadrupolar_/(*I*_Quadrupolar_ + *I*_Czjzek_) changes from 0.2 to 0.6 upon varying the ACP:flux ratio (from 1:1 to 1:10), indicating the formation of particles of higher crystallinity. Moreover, based on the SEM images (Figure S7, Supporting Information), it is seen that larger particles are formed.

**Figure 4:**
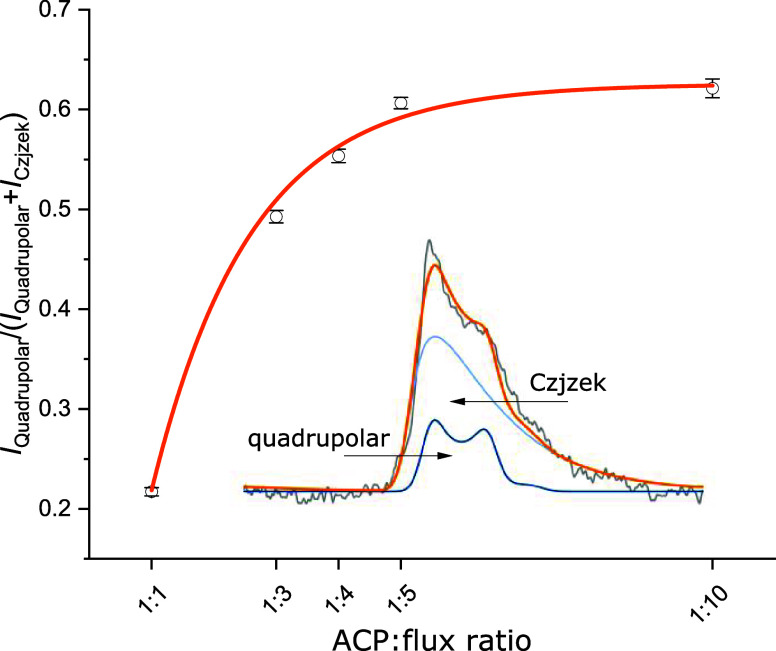
Dependence of the parameter *I*_Quadrupolar_/(*I*_Quadrupolar_ + *I*_Czjzek_) on the ACP:flux ratio used during the synthesis (KCl:CaCl_2_ = 9:1, *T* = 750 °C, *t* = 5 h). Error margins are within the circles indicating data points. The exemplary ^35^Cl MAS NMR spectrum obtained for the Ca_5_(PO_4_)_3_Cl sample (ACP:flux = 1:1), along with the fitted lines, is shown for clarity.

To investigate the effects of synthesis temperature on the phases formed, the ^35^Cl MAS and echo NMR measurements were performed for the set of samples synthesized with the following parameters ACP:flux ratio = 1:2, KCl:CaCl_2_ = 6:4, and treating temperature of 750–1200 °C (Supporting Information, Figures S8–S11). In addition, XRD patterns were obtained for these samples, indicating the formation of a monoclinic crystal structure.^22^ As it was previously determined, the ^35^Cl NMR spectra were fitted using lineshapes consisting of quadrupolar and Czjzek distribution components. It is seen that the quadrupolar lineshape parameters are independent of treatment temperature being *δ*_iso_ = 157 ppm, *C*_Q_ = 1.7 MHz, and *η* = 0. The isotropic chemical shift of the Czjzek lineshape is also independent of treatment temperature being *δ*_iso_ = 159 ppm, though the perturbation parameter *σ* increases with higher treatment temperature (*σ* = 0.4, 0.45, 0.5, and 0.55 for *T* = 900, 1000, 1100, and 1200 °C, respectively). This indicates a broadening of the distribution of chemical environments within the glasslike phase in the samples.

The potential of ^35,37^Cl solid-state NMR in studying complex inorganic chlorine-containing materials featuring several phases within was shown. ^35,37^Cl solid-state NMR was proven to be more sensitive than ^31^P solid-state NMR in distinguishing crystalline from glasslike species. Such a breakthrough may be further applied to studying chlorine-containing inorganic solids, especially those with more symmetric chlorine environments. Moreover, the potential of the proposed method should be further exploited to study other NMR-active nuclei, e.g., ^51^V, ^11^B, ^27^Al, ^23^Na, etc., and a variety of functional materials, such as light-, energy-, and biorelated materials or catalysts, featuring complex structures.

## 4 Conclusions

A phase composition in a series of Ca_5_(PO_4_)_3_Cl powders was studied using ^35^Cl and ^37^Cl MAS and echo NMR. In comparison to ^31^P MAS NMR, the ^35^Cl and ^37^Cl NMR have shown sensitivity in highlighting the crystalline and glasslike phases present in the samples. Particularly, the ^35^Cl and ^37^Cl NMR of Ca_5_(PO_4_)_3_Cl samples showed the presence of two spectral lines. The spectral components fitted using the quadrupolar lineshape model are assigned to the cores of particles possessing high crystallinity, with the following parameters: *δ*_iso_ = 157 ppm, *C*_Q_ = 1.7 MHz, and *η* = 0. Meanwhile, the spectral components fitted using the Czjzek model, with the following parameters: *δ*_iso_ = 159 ppm and *σ* = 0.33–0.55, are assigned to the glasslike structural phases primarily formed on the surface of the particles. This assignment was corroborated by TEM analysis.

In addition, to the best of our knowledge, this study reports the first ^35^Cl wide-line NMR spectrum of the spodiosite-type Ca_2_PO_4_Cl. The ^35^Cl static echo acquisition was performed in a piecewise manner, and the following quadrupolar line parameters were obtained: *δ*_iso_ = 106 ppm, *C*_Q_ = 7.9 MHz, and *η* = 0.2. Further analysis of such systems using WURST pulses and QCPMG experiments should enable acquisition of the ^35^Cl static NMR spectrum for Ca_2_PO_4_Cl without frequency stepping.^43^

## Supplementary Material


