# Supplementary material for: Chlorine Nuclear Magnetic Resonance as a Sensitive Probe to Study Crystalline to Glasslike Interfaces in Calcium Phosphates
Source: Langmuir. 2026 May 26;42(22):15505–11. doi: 10.1021/acs.langmuir.6c01029 (PMC13262052; doi:10.1021/acs.langmuir.6c01029)
Supplement: Supplementary file 1 [file la6c01029_si_001.pdf]

Supporting Information

Chlorine Nuclear Magnetic Resonance as a Sensitive Probe to Study Crystalline to Glasslike Interfaces in Calcium Phosphates

Rokas Lemežis[1], *Jonas Stadulis*[2], *Audrius Drabavičius*[3], *Aleksej Zarkov*[2],

Vytautas Klimavicius[1*]

[1]Institute of Chemical Physics, Vilnius University, Saulėtekio al. 3, LT-10257 Vilnius, Lithuania.

[2]Institute of Chemistry, Vilnius University, Saulėtekio al. 3, LT-10257 Vilnius, Lithuania.

[3]Center for Physical Sciences and Technology (FTMC). Saulėtekio al. 3, LT-10257, Vilnius, Lithuania.

Author to whom correspondence should be addressed:

Vytautas Klimavicius: e-mail vytautas.klimavicius@ff.vu.lt

Table S1. ^{35}Cl MAS and static NMR spectra fitting parameters obtained for $Ca_5(PO_4)_3Cl$ representative sample (K:Ca = 8:2, T = 750 °C, ACP:flux = 1:2, t = 5 h).

	Quadrupolar lineshape	Czjzek lineshape
δ_{iso}, ppm	157.7±0.2	159.1±0.5
C_Q, MHz	1.7±0.01	-
η_Q	0	-
σ, MHz	-	0.48±0.01

	9.4 T ^{35}Cl MAS NMR	14.1 T ^{35}Cl MAS NMR	9.4 T ^{35}Cl wideline NMR
$I_{Quadrupolar}/(I_{Quadrupolar}+I_{Czjzek})$	0.54±0.02	0.26±0.02	0.73±0.02
Gaussian apodization, Hz	313±38	259±51	1560±152
R^2	0.92	0.96	0.95

Table S2. ^{37}Cl MAS NMR fitting parameters obtained after initial fitting procedure performed for the $Ca_5(PO_4)_3Cl$ sample (K:Ca = 8:2, T = 750 °C, ACP:flux = 1:2, t = 5 h).

	Quadrupolar lineshape	Czjzek lineshape
δ_{iso}, ppm	162.1±0.4	161±1.2
C_Q, MHz	1.34±0.01	-
η_Q	0	-
σ, MHz	-	0.31±0.01

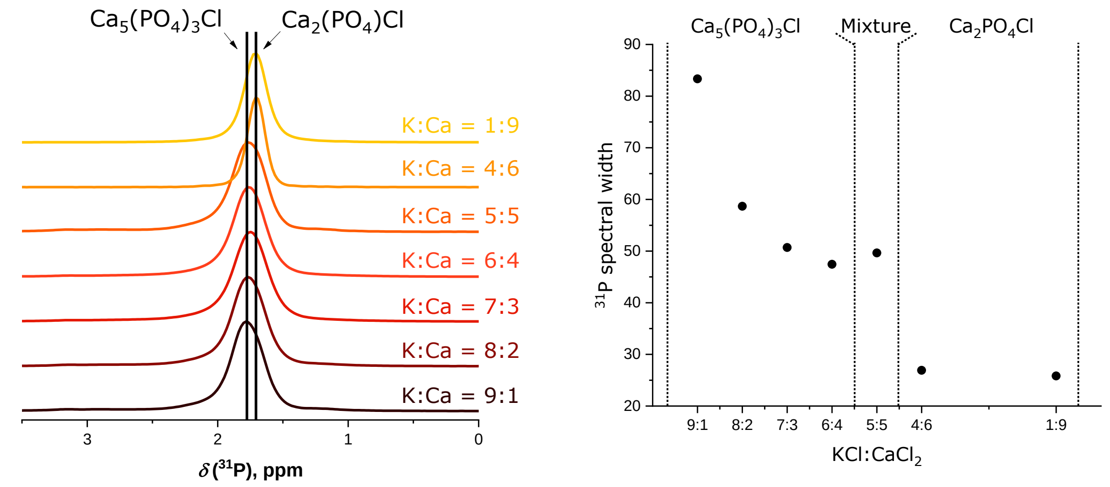

Figure S1. Left - ^{31}P MAS NMR spectra of $Ca_5(PO_4)_3Cl$ and Ca_2PO_4Cl samples (T = 750 °C, ACP/flux = 1:2, t = 5 h). Right - the ^{31}P MAS NMR line FWHM depending on $KCl:CaCl_2$ ratio. Note the FWHM of the ^{31}P MAS NMR line obtained for the sample indicated as mixture of $Ca_5(PO_4)_3Cl$ and Ca_2PO_4Cl ($KCl:CaCl_2$ = 5:5) corresponds to the sum of both lines.

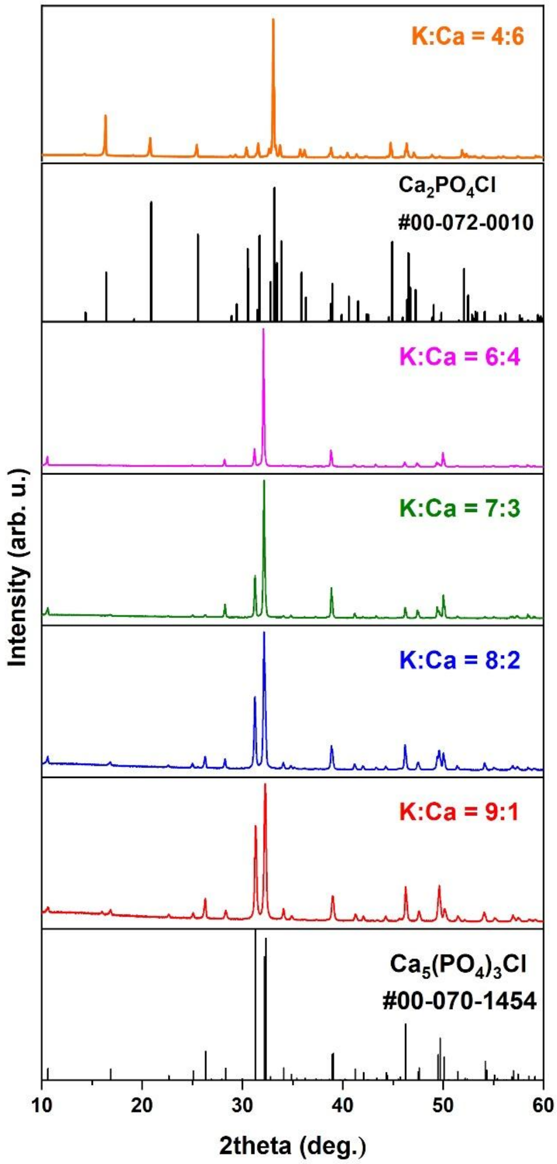

Figure S2. XRD patterns of the synthesis products obtained after the treatment of ACP in the mixtures of KCl and $CaCl_2$ mixed in different molar proportions (T = 750 °C, ACP:flux = 1:2, t = 5 h).

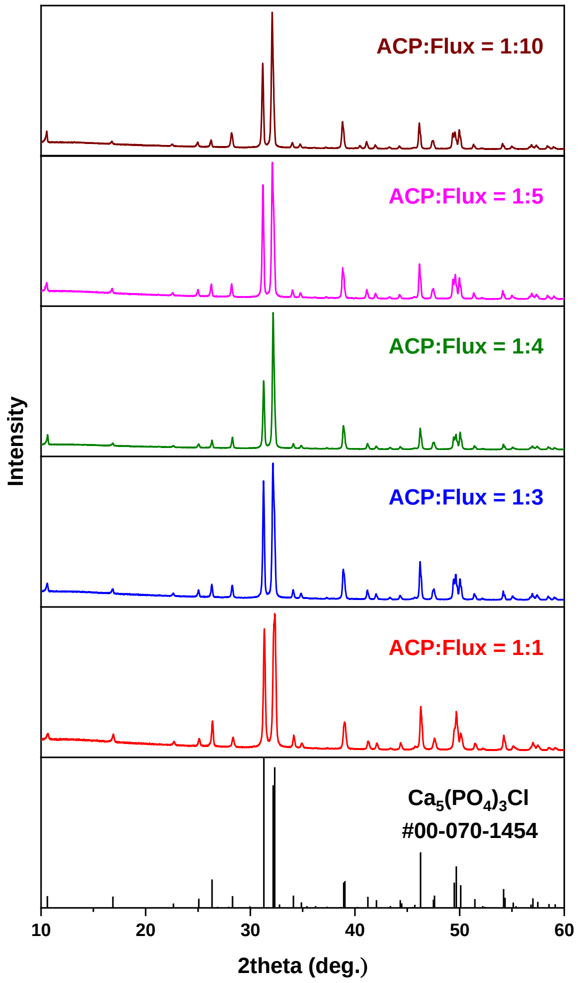

Figure S3. XRD patterns of the synthesis products with various ACP:flux ratios used during the synthesis (KCl:$CaCl_2$ = 9:1, T = 750 °C, t = 5 h).

^{35}Cl static NMR measurements of spodiosite-type Ca_2PO_4Cl

Sample containing spodiosite-type (Goryainovite) Ca_2PO_4Cl phase was analyzed using ^{35}Cl NMR and piecewise acquisition at 9.4 T using a 5 mm wideline NMR probe. Temperature was stabilized at 298 K, π/2 pulse was 4 μs (62.5 kHz excitation width) and recycle delay was set to 1 s. Acquisition was performed in a piecewise manner with 60 kHz excitation offset, in total 7 subspectra were measured which were later summed-up (**Figure S4**). Solid-echo pulse sequence was used (π/2 - delay - π/2 - delay - acquire) with 50 μs interpulse delay and 20480 scans were accumulated for each subspectra.

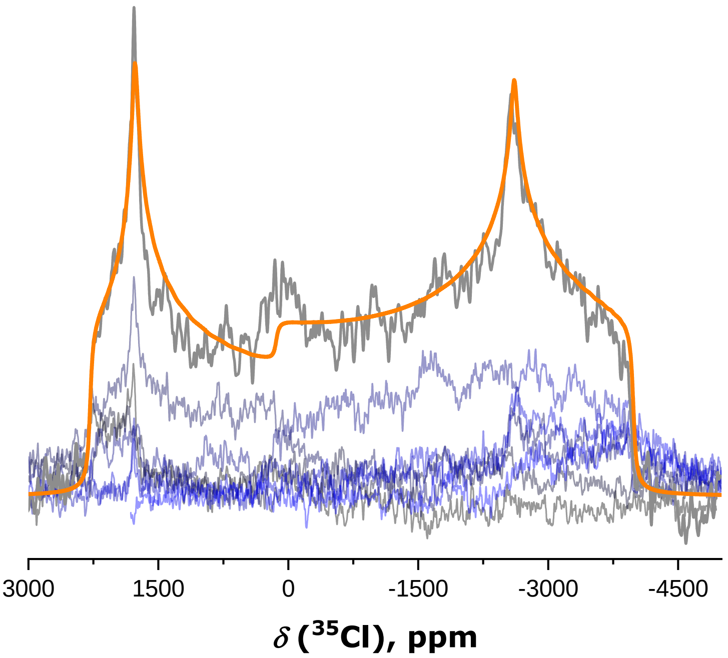

Figure S4. ^{35}Cl static NMR spectrum measured using piecewise acquisition obtained for Goryainovite Ca_2PO_4Cl sample synthesized using following parameters: KCl:$CaCl_2$ = 4:6, T = 750 °C, ACP/flux = 1:2, t = 5 h. Parameters of quadrupolar model fit are δ_{iso} = 106 ppm, C_Q = 7.9 MHz, η = 0.2. Sum of all sub-spectra is shown in grey, spectral fitting in orange color.

$^{35,37}Cl$ NMR of $Ca_5(PO_4)_3Cl$ samples with varying KCl:$CaCl_2$ ratio in the flux during synthesis.

$Ca_5(PO_4)_3Cl$ samples with synthesis parameters of KCl:$CaCl_2$ = 9:1; 8:2; 7:3; 6:4, T = 750 °C, ACP:flux = 1:2 and t = 5 h were analysed using ^{35}Cl NMR (**Table S3**). The signal consisted of the overlapped quadrupolar and *Czjzek* model lineshapes with the following parameters: δ_{iso} = 157 ppm, C_Q = 1.7 MHz, η = 0 for quadrupolar and δ_{iso} = 159 ppm, σ = 0.4 Mhz for *Czjzek* lineshapes. As it was previously mentioned, the difference in KCl:$CaCl_2$ ratio does not influence lineshape parameters and in turn the chemical environment of chlorine nuclei.

Table S3. ^{35}Cl NMR spectral fitting results of $Ca_5(PO_4)_3Cl$ samples.

Synthesis parameters				^{35}Cl modeling parameters				
				Quadrupolar lineshape			Czjzek lineshape	
$KCl:CaCl_2$	T, ˚C	Time, h	ACP/flux	δ_{iso}, ppm	C_Q, MHz	η_Q	δ_{iso}, ppm	σ, MHz
9:1	750	5	1:2	157	1.7	0	159	0.4
8:2	750	5	1:2	157	1.7	0	159	0.4
7:3	750	5	1:2	157	1.7	0	159	0.4
6:4	750	5	1:2	157	1.7	0	159	0.4
9:1	750	5	1:1	157	1.7	0	159	0.5
9:1	750	5	1:3	157	1.7	0	159	0.5
9:1	750	5	1:4	157	1.7	0	159	0.5
9:1	750	5	1:5	157	1.7	0	159	0.5
9:1	750	5	1:10	157	1.7	0	159	0.5
6:4	900	5	1:2	157	1.7	0	159	0.4
6:4	1000	5	1:2	157	1.7	0	159	0.45
6:4	1100	5	1:2	157	1.7	0	159	0.5
6:4	1200	5	1:2	157	1.7	0	159	0.55

Table S4. ^{35}Cl MAS NMR spectral line intensities and Gaussian apodization parameters of $Ca_5(PO_4)_3Cl$ at different magnetic fields.

Synthesis parameters				Model parameters					
				9.4 T ^{35}Cl MAS NMR			14.1 T ^{35}Cl MAS NMR		
$KCl:CaCl_2$	T, ˚C	Time, h	ACP/flux	$I_{Quadrupolar}/(I_{Quadrupolar}+I_{Czjzek})$	Gaussian apodization, Hz	R^2	$I_{Quadrupolar}/(I_{Quadrupolar}+I_{Czjzek})$	Gaussian apodization, Hz	R^2
9:1	750	5	1:2	0.07±0.01	500	0.91	0.13±0.05	200	0.92
8:2	750	5	1:2	0.56±0.01	450	0.95	0.38±0.03	200	0.95
7:3	750	5	1:2	0.87±0.02	450	0.95	0.67±0.04	200	0.91
6:4	750	5	1:2	0.59±0.01	450	0.91	0.71±0.03	200	0.88
9:1	750	5	1:1	0.22±0.01	750	0.98	-	-	-
9:1	750	5	1:3	0.49±0.01	750	0.97	-	-	-
9:1	750	5	1:4	0.55±0.01	750	0.96	-	-	-
9:1	750	5	1:5	0.61±0.01	750	0.97	-	-	-
9:1	750	5	1:10	0.62±0.01	750	0.97	-	-	-
6:4	900	5	1:2	0.74±0.01	450	0.93	0.55±0.03	200	0.95
6:4	1000	5	1:2	0.55±0.02	450	0.9	-	-	-
6:4	1100	5	1:2	0.49±0.02	450	0.96	0.39±0.08	200	0.93
6:4	1200	5	1:2	0.29±0.01	450	0.92	0.35±0.04	200	0.94

Table S5. ^{37}Cl MAS NMR spectral fitting results obtained for $Ca_5(PO_4)_3Cl$ samples.

Synthesis parameters				^{37}Cl modeling parameters				
				Quadrupolar lineshape			Czjzek lineshape	
$KCl:CaCl_2$	T, ˚C	Time, h	ACP/flux	δ_{iso}, ppm	C_Q, MHz	η_Q	δ_{iso}, ppm	σ, MHz
9:1	750	5	1:2	160	1.35	0	163	0.33
8:2	750	5	1:2	160	1.35	0	163	0.33
7:3	750	5	1:2	160	1.35	0	163	0.33
6:4	750	5	1:2	160	1.35	0	163	0.33
6:4	900	5	1:2	160	1.35	0	163	0.33
6:4	1000	5	1:2	160	1.35	0	163	0.33
6:4	1100	5	1:2	160	1.35	0	163	0.33
6:4	1200	5	1:2	160	1.35	0	163	0.33

Table S6. ^{35}Cl static and ^{37}Cl MAS NMR spectral line intensities and Gaussian apodization parameters obtained for the $Ca_5(PO_4)_3Cl$ samples.

Synthesis parameters				Model parameters					
				9.4 T ^{35}Cl echo NMR			9.4 T ^{37}Cl MAS NMR		
$KCl:CaCl_2$	T, ˚C	Time, h	ACP/flux	$I_{Quadrupolar}/(I_{Quadrupolar}+I_{Czjzek})$	Gaussian apodization, Hz	R^2	$I_{Quadrupolar}/(I_{Quadrupolar}+I_{Czjzek})$	Gaussian apodization, Hz	R^2
9:1	750	5	1:2	0.13±0.01	1400	0.95	0.17±0.05	400	0.93
8:2	750	5	1:2	0.68±0.01	1400	0.9	0.27±0.05	400	0.92
7:3	750	5	1:2	0.85±0.02	1400	0.9	0.88±0.04	400	0.94
6:4	750	5	1:2	0.61±0.01	1400	0.97	0.85±0.05	400	0.97
6:4	900	5	1:2	0.79±0.03	1400	0.82	0.65±0.04	400	0.95
6:4	1000	5	1:2	0.76±0.02	1400	0.89	0.54±0.04	400	0.96
6:4	1100	5	1:2	0.55±0.01	1400	0.9	0.55±0.05	400	0.96
6:4	1200	5	1:2	0.34±0.01	1400	0.97	0.52±0.04	400	0.96

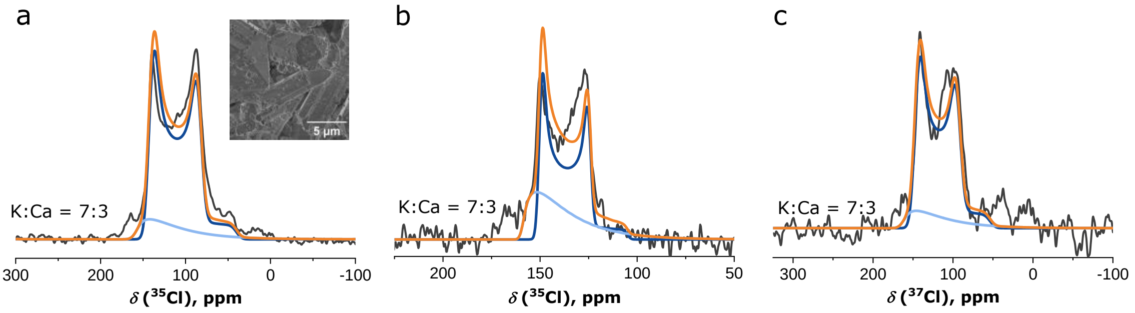

Figure S5. ^{35}Cl MAS NMR spectra of $Ca_5(PO_4)_3Cl$ obtained at 9.4 T (a) and 14.1 T (b) magnetic fields, and ^{37}Cl MAS NMR spectrum obtained at 9.4 T (c). $Ca_5(PO_4)_3Cl$ sample synthesised with the following parameters: $KCl:CaCl_2$ = 7:3, T = 750 °C, ACP/flux = 1:2, t = 5 h). Spectral fitting is shown in dark blue - quadrupolar, in light blue - *Czjzek* models and total fitting in orange. SEM micrograph is shown for reference.

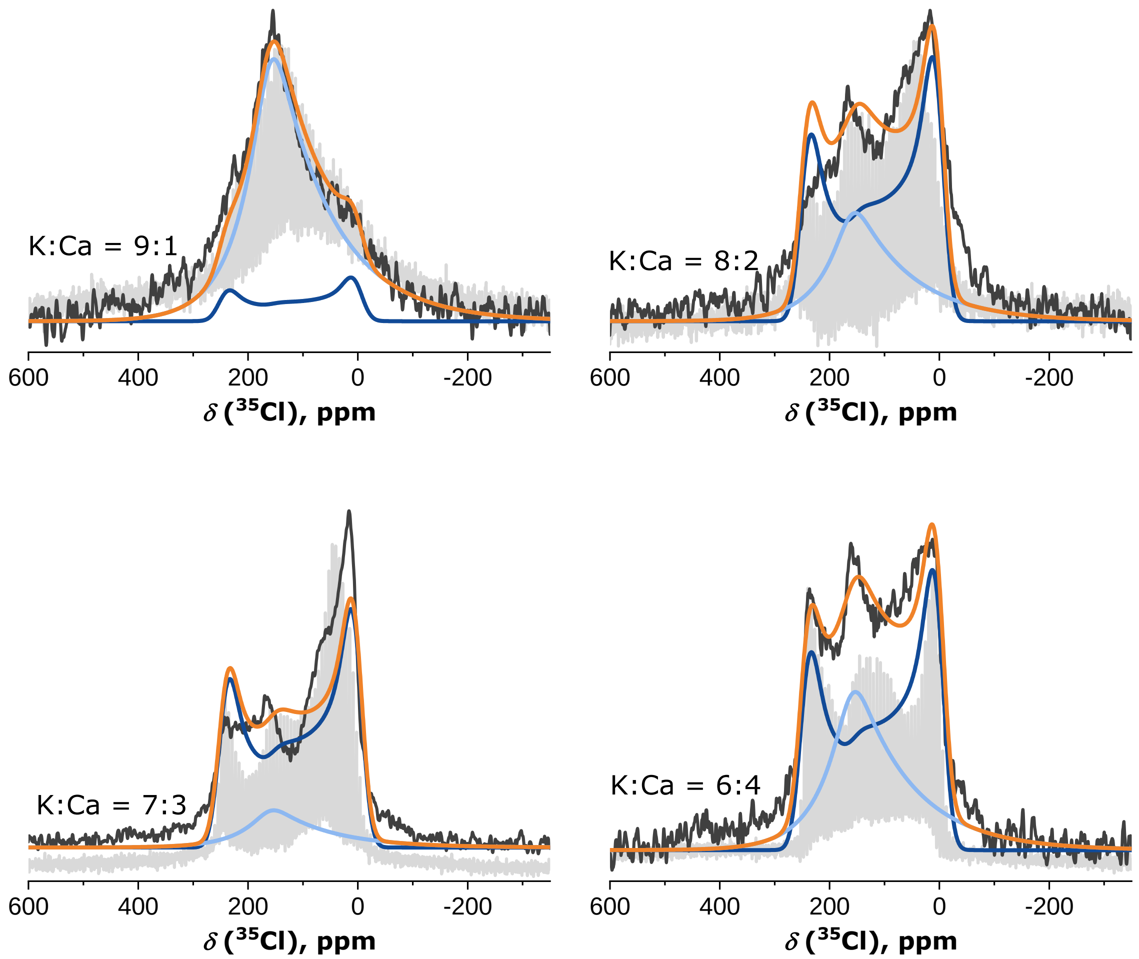

Figure S6. ^{35}Cl static echo and ^{35}Cl QCPMG NMR spectra of $Ca_5(PO_4)_3Cl$ samples synthesized using different molar ratio K:Ca in the flux (from 9:1 to 6:4, T = 750 °C, ACP/flux = 1:2, t = 5 h). Spectral fitting is shown in dark blue - quadrupolar, in light blue - *Czjzek* models and total fitting in orange.

^{35,37}Cl NMR of $Ca_5(PO_4)_3Cl$ samples with varying ACP:flux ratio during synthesis ($KCl:CaCl_2$ = 9:1).

$Ca_5(PO_4)_3Cl$ samples with synthesis parameters of $KCl:CaCl_2$ = 9:1, T = 750 °C, t = 5 h and ACP:flux = 1:1; 1:3; 1:4; 1:5; 1:10 possessed ^{35}Cl MAS NMR signal consisting of overlapped quadrupolar and *Czjzek* model lineshapes. Variation of ACP:flux ratio does not influence lineshape parameters which are δ_{iso} = 157 ppm, C_Q = 1.7 MHz, η = 0 for quadrupolar and δ_{iso} = 159 ppm, σ = 0.5 MHz for *Czjzek* lineshapes. Relative integral intensities and R^2 parameters are given in **Table S4**.

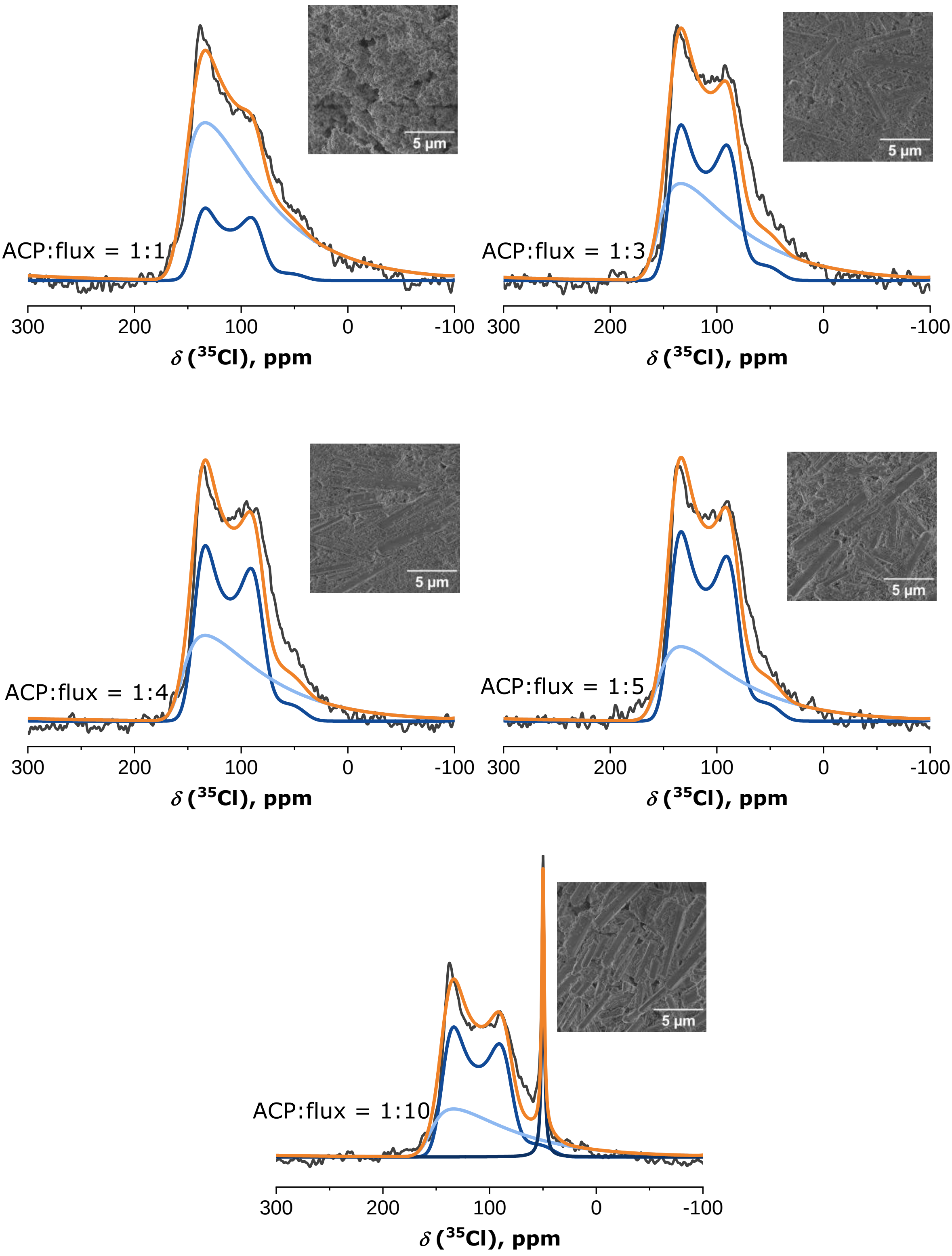

Figure S7. ^{35}Cl MAS NMR spectra of $Ca_5(PO_4)_3Cl$ samples synthesized by varying ACP:flux ratio (KCl:$CaCl_2$ = 9:1, T = 750 °C, t = 5 h). Spectral fitting is shown in dark blue - quadrupolar, in light blue - *Czjzek* models and total fitting in orange. SEM micrographs shown for reference.

Annealing temperature effects on the ^{35}Cl MAS NMR spectra

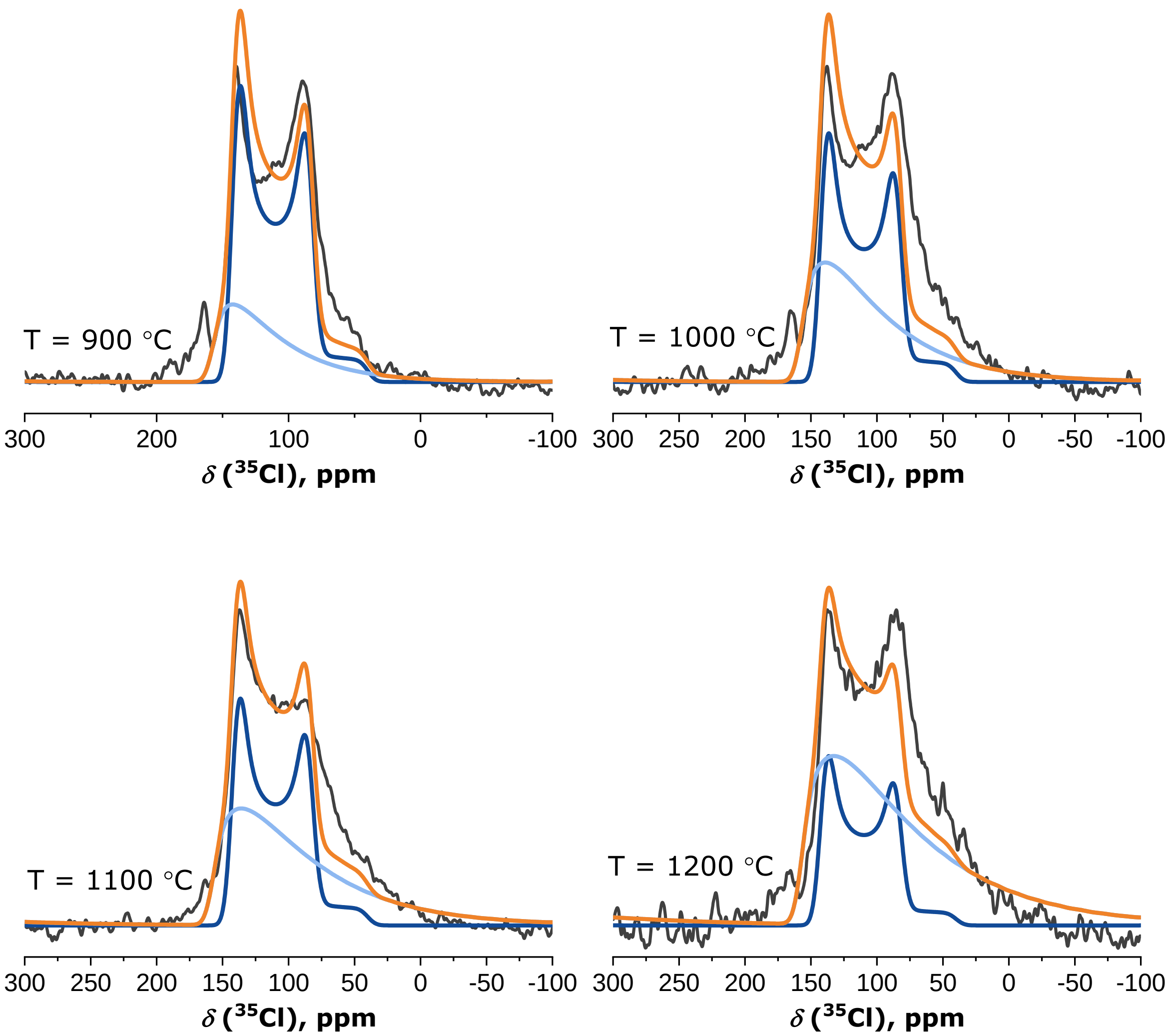

Figure S8. ^{35}Cl MAS NMR spectra obtained at 9.4 T for $Ca_5(PO_4)_3Cl$ samples synthesized by varying annealing temperature during synthesis (KCl:$CaCl_2$ = 6:4, ACP:flux = 1:2, t = 5 h). Spectral fitting is shown in dark blue - quadrupolar, in light blue - *Czjzek* models and total fitting in orange.

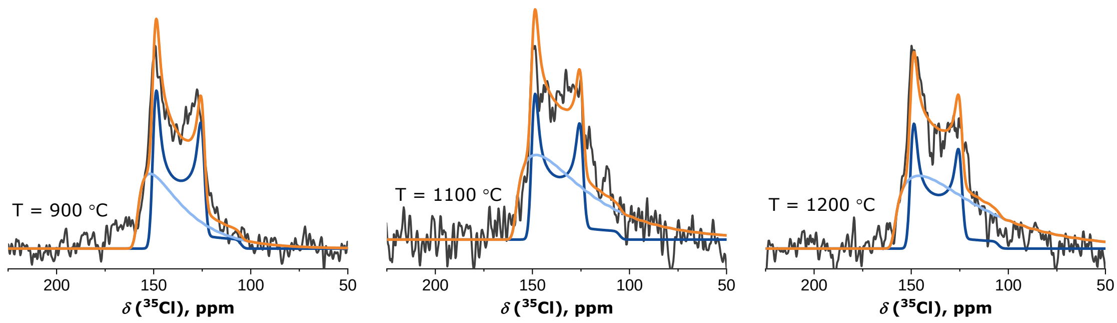

Figure S9. ^{35}Cl MAS NMR spectra obtained at 14.1 T magnetic field for $Ca_5(PO_4)_3Cl$ samples synthesized by varying annealing temperature during synthesis ($KCl:CaCl_2$ = 6:4, ACP:flux = 1:2, t = 5 h). Spectral fitting is shown in dark blue - quadrupolar, in light blue - *Czjzek* models and total fitting in orange.

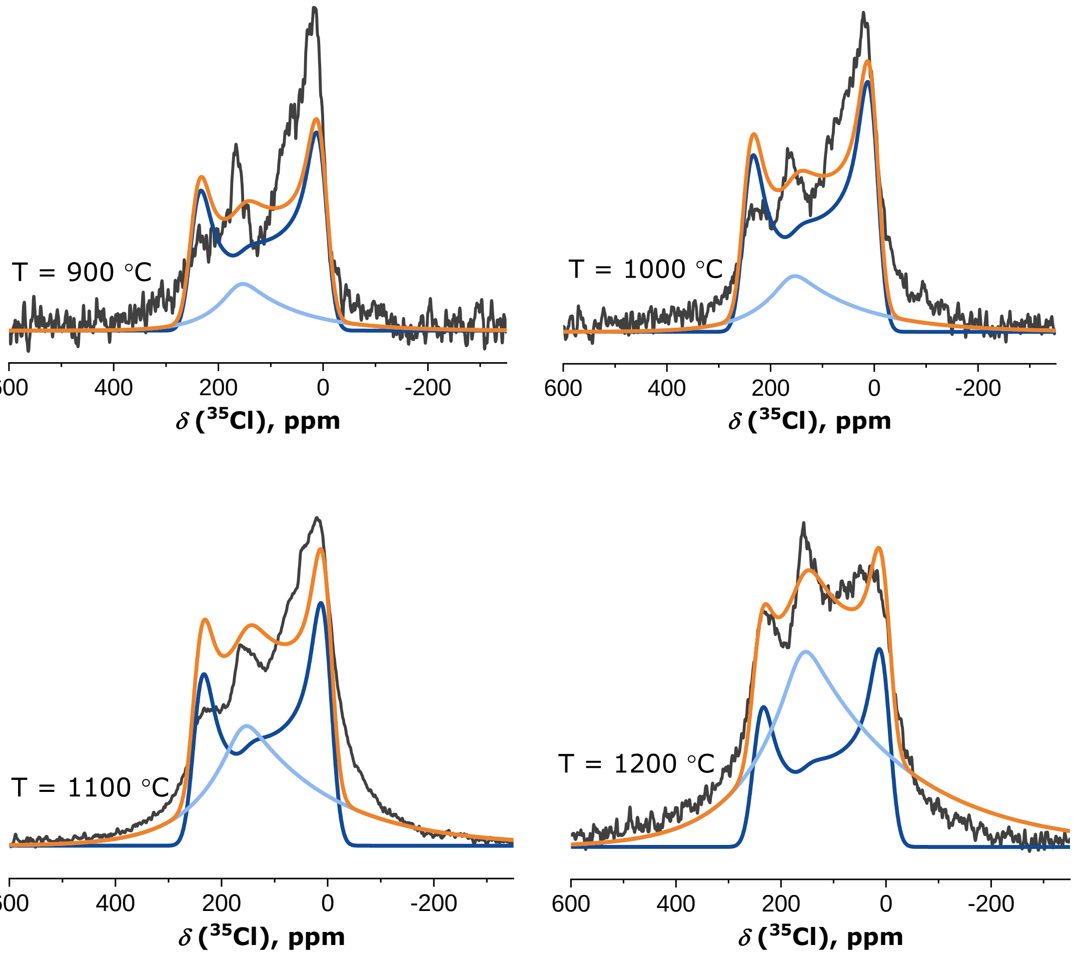

Figure S10. ^{35}Cl static echo NMR spectra obtained at 9.4 T for $Ca_5(PO_4)_3Cl$ samples synthesized by varying annealing temperature during synthesis ($KCl:CaCl_2$ = 6:4, ACP:flux = 1:2, t = 5 h). Spectral fitting is shown in dark blue - quadrupolar, in light blue - *Czjzek* models and total fitting in orange.

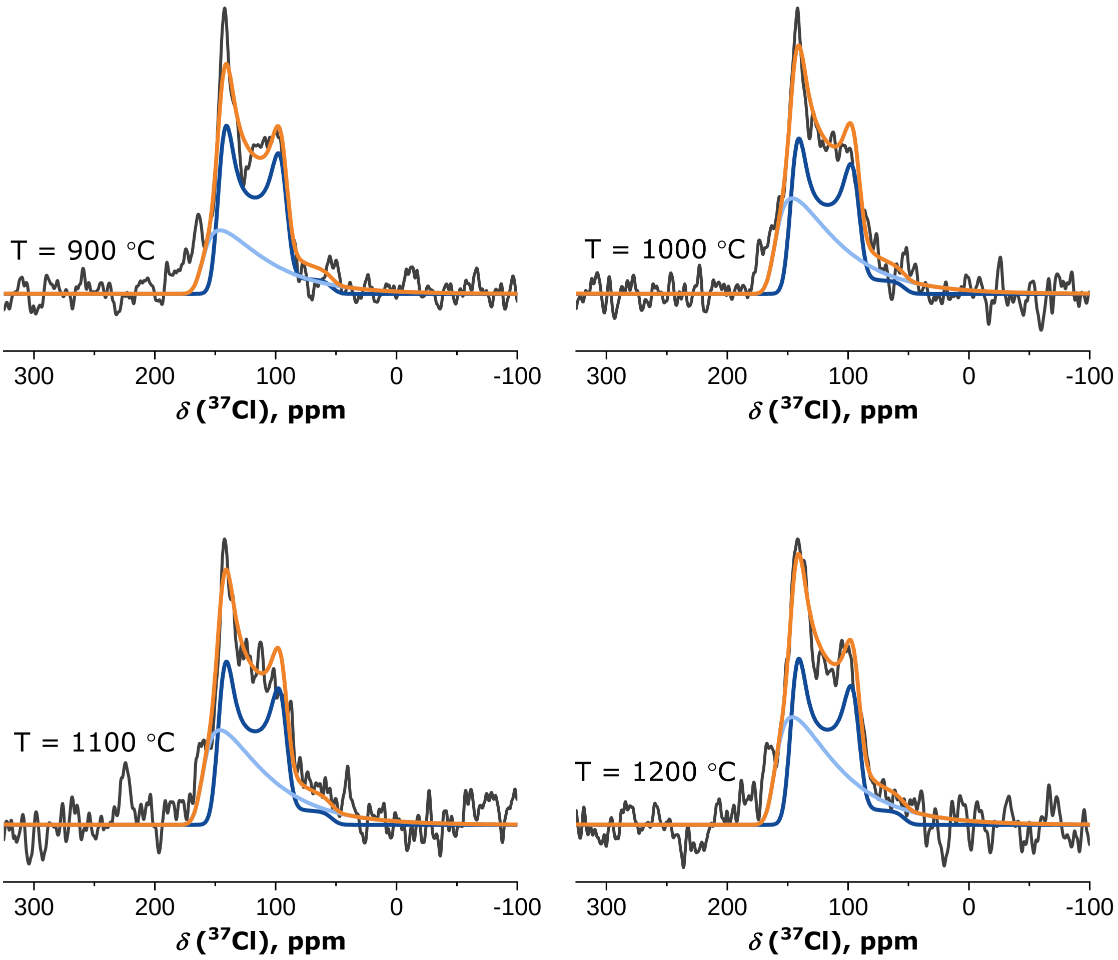

Figure S11. ^{37}Cl MAS NMR spectra obtained at 9.4 T for $Ca_5(PO_4)_3Cl$ samples synthesized by varying annealing temperature during synthesis (KCl:$CaCl_2$ = 6:4, ACP:flux = 1:2, t = 5 h). Spectral fitting is shown in dark blue - quadrupolar, in light blue - *Czjzek* models and total fitting in orange.
